# Metacarpal Bone Plane Examination by Ultrasonography for the Diagnosis of Fetal Forearm and Hand Deformity

**DOI:** 10.1038/srep42161

**Published:** 2017-02-07

**Authors:** Leiping Zhou, Mingli Lv, Min Zeng, Yun Zhou, Tian Yang, Yu Yang, Yunyun Cao, Xiaoxiao Kong, Jianmei Niu

**Affiliations:** 1 Ultrasound Department, The International Peace Maternity & Child Health Hospital of China Welfare Institute, 200030, Shanghai, China.

## Abstract

We explored the value of the metacarpal bone plane in screening for serious fetal forearm and hand deformities, excluding simple polydactyly and dactylion deformity, by ultrasonographic examination. Observed the second to fifth metacarpal bone plane of fetuses in 20,139 pregnant women at a gestational age of 16 to 30 weeks in The International Peace Maternity & Child Health Hospital of China Welfare Institute (IPMCH). There was a total 138 cases of fetal forearm and/or hand deformity among the 20,139 pregnant women. Of these, 134 cases were diagnosed, 4 cases were not diagnosed, and 1 case was misdiagnosed. Among the 134 diagnosed cases, there were 19 cases of hand absence, 5 cases of cleft hand, 13 cases of ectrodactyly, 26 cases of radius absence, 9 cases of forearm and hand dysplasia, 55 cases of thanatophoric dysplasia, 6 cases of wrist joint dysplasia, and 1 case of forearm amputation deformity. The deformity rate was 0.76%, the diagnostic coincidence rate was 99.97%, the sensitivity was 97.10%, the specificity was 99.99%, and the false negative rate was 2.9%. As such, careful observation of the metacarpal bone plane can be used increase the diagnosis rate of fetal forearm and hand deformity.

With recent advances in ultrasound technology, both the prevalence and accuracy of second-trimester sonography have increased over the past decade. In most countries, fetal anomaly screens have become routine examinations for pregnant women at a gestational age of 24 to 26 weeks.

Serious fetal forearm and hand deformities, although not fatal, can have a strong impact on family and social relationships if they are not diagnosed before birth. According a report by Xu Heng (in Chinese)^1^, missed diagnoses of hand and/or foot deformity comprised a large proportion of medical disputes caused by missed or misdiagnosed ultrasonography for prenatal diagnosis. Up to two-thirds of limb abnormalities detected in the second and third trimesters are associated with serious or fatal disorders^2^,^3^,^4^,^5^,^6^. However, most of the reports in the literature are case reports or only include a small sample size.

Our study aimed to explore the value of examining the metacarpal bone plane using ultrasonography in screening for serious fetal forearm and hand deformities, excluding simple polydactyly and dactylion deformity. The work was designed to identify a method that would improve the accuracy of diagnosing serious forearm and hand deformities, while being quickly, easily, and conveniently implementable in clinical settings.

## Materials and Methods

### Basic information

Ultrasonographic fetal examinations were performed on 20,139 pregnant women at gestational ages of 16 to 30 weeks in the IPMCH from June 2008 to June 2015. The pregnant women ranged in age from 21 to 48 years old, and the median age was 31 years. All the newborns, whether by natural or induced labor, were followed-up. Our study was approved by the affiliated IPMCH ethics committee. All the examinations were performed in accordance with the requirements of the ethics committee and the prenatal ultrasonographic diagnosis guide, which was promulgated by the Chinese medical doctor association in 2012. Before examination, written informed consent was obtained from all the pregnant women that were recruited to our study.

### Equipment and method

Equipment: GE Voluson E8, GE Voluson E6, GE Voluson 730 Expert, and Philips HD11DX color doper ultrasonoscopes were used. The transducer frequency was set to 3.5 to 5.0 MHz.

Method: The terminal region of the forearm and the second to fifth metacarpal bone plane were carefully observed after routine examination of the fetus, amniotic fluid and placenta. First, the long axes of the humerus, ulna and radius were observed. Then, as the distal radius and ulna at the fulcrum were approached, the axis was rotated to clearly show the second to fifth metacarpal plane. If the obtained images were not satisfactory, we advised the pregnant women to walk around for 20 minutes, after which time we repeated the examination.

### Observation index

Generally, the four metacarpal bones (second to fifth) should all occur in one plane, be fan-shaped, and have no crossing or overlap. Specifically, we observed the number, length, morphology, and spacing of the metacarpal bone planes, along with the angles between them.

### Statistical analysis

A fourfold table chi-square test was using to evaluate the sensitivity, specificity, precision rate (positive rate) and missed diagnosis rate of the method.

## Result

### Serious fetal hand deformities

Among the 20,139 pregnant women, we diagnosed 134 cases of serious fetal hand deformity, excluding simple polydactyly and dactylion deformity. Of these, 51 cases were born or aborted at our hospital, while 83 cases occurred at other hospitals and were confirmed by telephone. In total, 134 cases showed consistency between prenatal ultrasound diagnosis and diagnosis after birth or abortion. They included 19 cases of hand absence 5 cases of cleft hand, 13 cases of ectrodactyly with abnormal finger morphology, 26 cases of radius absence or hypoplasia, 9 cases of forearm and hand dysplasia, 55 cases of thanatophoric dysplasia, 6 cases of wrist joint dysplasia, and 1 case of forearm amputation deformity.

In 4 cases, diagnosis was missed, including 1 case of left cleft hand, 1 case of two-finger (thumb and little finger) absence on the left hand, 1 case of overlapping fingers of two hands, and 1 case of missing dactylus in the index finger and middle finger. Only 1 case was misdiagnosed, and this was because of a low level of amniotic fluid that was caused by a urinary system deformity. After abortion, we found that there was no obvious deformity in the fetal arm or hand.

**Figure 1:**
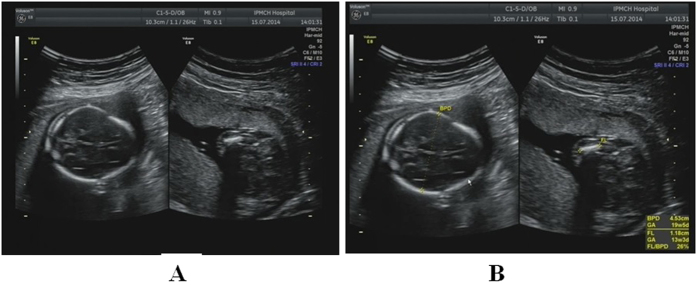
Thanatophoric dysplasia at a gestational age of 18 weeks. (**A**) Two-dimensional sonogram showing a biparietal diameter (BPD) of 45 mm and a thighbone length of 12 mm, indicating the shortening of all limbs. (**B**) The estimated gestational age of the fetus was 19 weeks and 5 days according to the BPD but 13 weeks and 3 days according to the length of the thighbone. The thighbone/BPD ratio was 26%.

All the serious fetal hand deformities that were detected are described as follows:

Thanatophoric dysplasia: A total of 55 cases were observed. The ultrasonoscopic properties included a shortening of the limbs (micromelia), where the thigh bone and humerus were more than 4 standard deviations shorter than the average length according to the week of gestation; a small conical thorax; and an obvious shortening of the second to fifth metacarpal bones (Fig. 1).

**Figure 2:**
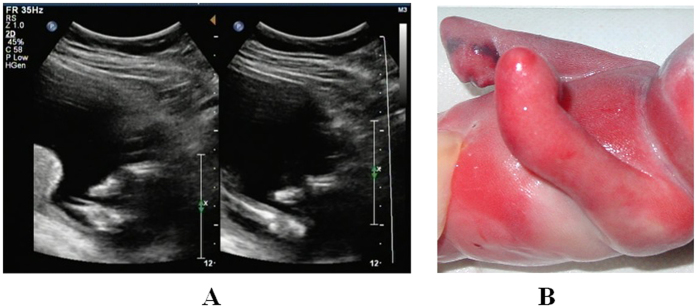
The absence of both radius was observed at a gestational age of 25 weeks. (**A**) Two-dimensional sonogram showing that both forearms are shortened, with only one long bone visible and with abnormal hand positioning (hook-like). The metacarpal bone plane could not be identified. (**B**) Macroscopic view of the fetus showing the shortening and bending of both arms. Additionally, both hands were in radius flexion, and abnormalities in the palm and fingers were observed.

Radius absence or hypoplasia: a total of 26 cases were observed. The ultrasonoscopic properties included radius absence or hypoplasia; the hand being in radius flexion, appressed and clinging to the medial border of the forearm, appearing hook-like, and repeatedly maintaining this posture for long periods; association with hand abnormalities of different extents; and thumb absence or aplasia (Fig. 2).

**Figure 3:**
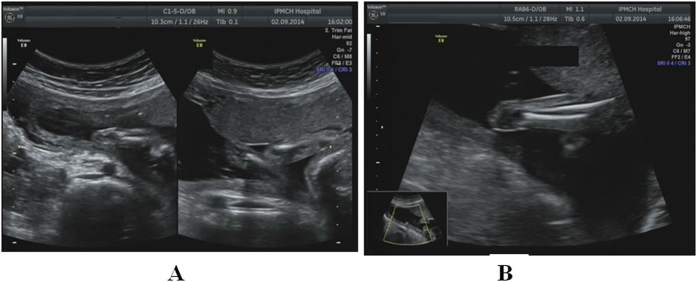
Absence of both hands at a gestational age of 22 weeks. (**A**) The metacarpal bone plane and fingers were invisible at the termini of both forearms, and the wrists were blind-ended. (**B**) Two-dimensional sonogram showing the ulna and radius in the right forearm, but the metacarpal bone plane and fingers cannot visible at any time.

Hand absence: a total of 19 cases were observed. The ultrasonoscopic properties included complete hand absence, with the absence of visible metacarpals and phalanges at the terminal region of the upper limb on longitudinal scanning; some cases showed small, dotted circular high-echoes (Fig. 3).

**Figure 4:**
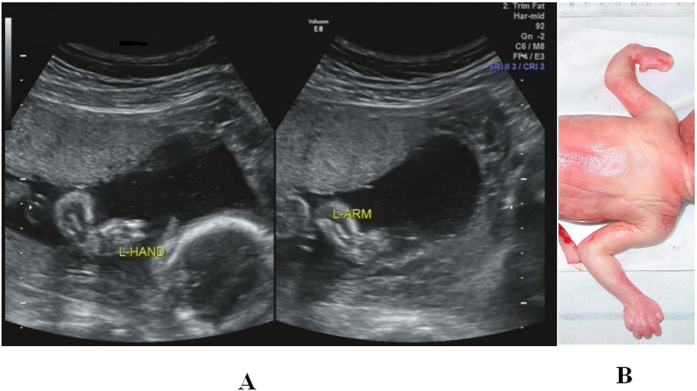
Left hand ectrodactyly at a gestational age of 24 weeks. (**A**) Two-dimensional sonogram showing the absence of some metacarpal bones in the fetal left hand. (**B**) Macroscopic view of the fetus, showing shorter fingers and palm abnormalities of the left hand.

Ectrodactyly: a total of 13 cases were observed. The ultrasonoscopic properties included the absence of some digits (one or more) and the absence of some associated metacarpals, leading to partial palm defects and irregularity (Fig. 4).

Wrist dysplasia: a total of 11 cases were observed. The ultrasonoscopic properties included abnormal hand positioning, wrist overbending to volar, the angle between the metacarpal plane and the forearm being less than 45°, and the wrist maintaining such a posture during examination, without moving.

Distal forearm and hand absence: a total of 9 cases were observed. The ultrasonoscopic properties included partial or complete invisibility of the radius and ulna, similar to upper extremity amputation, with the distal upper limb and hand not being visible.

Cleft hand: a total of 5 cases were observed. The ultrasonoscopic properties showed three types, specifically typical V-shaped clefting with the presence of metacarpals and typical crab-clawing of the extreme fingers; atypical cleft, which was often U- or spread U-shaped, involved one limb, and showed partial/complete absence of metacarpals and hypoplastic thumb and little finger; and Nil-clefting, in which all of the digits were absent and there was no further cleft.

Forearm amputation deformity: only 1 case was observed. The ultrasonoscopic properties included the complete absence of the forearm, radius and ulna and the hand being connected to the terminal region of the upper arm and humerus.

The sensitivity, specificity, diagnostic coincidence rate and false-negative rate of this method for detecting severe forearm and hand abnormalities by observing the second to fifth metacarpal plane were 97.10%, 99.99%, 99.97%, and 2.90%, respectively (Table 1).

### Associated abnormalities

In all 138 cases, including the 4 cases in which diagnosis was missed, 42 cases had abnormalities in other systems, including the heart, central nervous system, spine, urinary system, digestive system, hydrocephalus, cheilopalatognathus, and acromphalus.

## Discussion

Limbs are traditionally assessed during pregnancy as markers of fetal growth, nutrition, and gestational age. However, the assessment of fetal limbs and the evaluation of deformities may also aid in the diagnosis of various chromosomal and non-chromosomal conditions and narrow the differential diagnosis in cases where associated abnormalities have also been identified^4^,^7^.

The causes of limb abnormalities are varied, and include chromosomal, syndromal, and idiopathic reasons. Limb abnormalities are among the most distinctive findings in aneuploid trisomy, including trisomy 18, trisomy 13, and trisomy 21^7^,^8^,^9^. Radial aplasia, limb-body wall complex, amniotic band syndrome, oligohydramnios deformation sequence, and many other syndromes also can cause or be associated with limb abnormalities^10^,^11^,^12^. Some teratogens, including warfarin^9^, ethylene glycol monomethyl ether and its metabolite 2-methoxyacetic acid^13^, and dofetilide^14^ can also lead to limb abnormalities.

In fetal anomaly screens, observation of the upper limbs is mainly limited to the humerus, ulna and radius. Additionally, the data collected and the methods used for hand examinations are often not standardized. The diagnosis of hand deformities is the most easily missed, and misdiagnosis of limb abnormalities often occurs. Therefore, we sought to develop a sonographic method to detect serious fetal hand deformities. We found that observation of the metacarpal bone plane can help in the identification of some serious hand deformities, excluding simple polydactyly and dactylion deformity. As such, the aim of this study was to explore the value of metacarpal bone plane observation using ultrasonography for the screening of serious fetal hand deformity.

The results showed that 134 cases of serious fetal hand deformity, excluding simple polydactyly and dactylion deformity, were diagnosed among the 20,139 pregnant women studied. Four cases were not diagnosed, and 1 case was misdiagnosed. The sensitivity, specificity, diagnostic coincidence rate, and false negative rate of the method were 97.10%, 99.99%, 99.97%, and 2.90%, respectively. Of the 134 cases, 51 were born or aborted at our hospital, while 83 cases occurred in other hospitals, and the diagnoses were confirmed by telephone. In 134 cases, the prenatal ultrasound diagnosis and the diagnosis after birth or abortion were concordant. These included 19 cases of abnormal hand absence, 5 cases of cleft hand, 13 cases of ectrodactyly with abnormal finger morphology, 26 cases of radius absence or hypoplasia, 9 cases of forearm and hand dysplasia, 55 cases of thanatophoric dysplasia, 6 cases of wrist joint dysplasia, and 1 case of forearm amputation deformity.

We conclude that it is feasible to use the metacarpal bone plane to detect serious fetal hand deformity. First, the rate at which the metacarpal bone plane is visible is high. In the second trimester, the mobility of the fetus is high, and there is relatively more amniotic fluid. As such, most fetal metacarpal bone planes can be easily detected. Second, the metacarpal bone plane can be quickly and easily observed, as it only requires rotation of the transducer at the fulcrum of the distal ulna and radius to obtain a satisfactory metacarpal bone plane image. The total time of this operation is less than 3 minutes. Third, serious forearm and hand abnormalities, with the exception of simple polydactyly and dactylion deformity, are often accompanied by abnormalities of the metacarpal bone plane. Such abnormalities include metacarpal bone absence and a decrease or shortening of the angle between the metacarpal bone and the forearm. The deformities that may be detected include thanatophoric dysplasia, radius absence or hypoplasia, forearm and hand dysplasia, cleft hand, ectrodactyly with abnormal finger morphology, wrist joint dysplasia, and forearm amputation deformity, among others. In our study, 134 of the 138 cases had metacarpal bone plane abnormalities, and the sensitivity of the technique was 97.10%.

However, some cases were still missed or misdiagnosed by this method. Such cases may have occurred for various reasons. First, this method may have limitations in detecting slight finger abnormalities, such as the absence of the middle phalanx in some fingers. Such an absence occurred in the case of missed diagnosis in this study, where the middle phalanges of the index finger and middle finger were absent. Second, many factors can influence the observance of fetal hands and may obscure certain features. For example, oligohydramnios can be sheltered by the fetal body or the uterine wall. In our study, we misdiagnosed one case with overlapping fingers of two hands and one case with the absence of two fingers in the left hand because the metacarpal bone was not visible due to oligohydramnios and obstruction by the uterine wall. Another misdiagnosed case also occurred due to oligohydramnios. Third, the sonographer may neglect the hand while searching for other serious fetal abnormalities.

Consensus has not yet been achieved regarding the time at which forearm and hand abnormalities should be diagnosed. Most clinicians choose the second trimester, during which fetal anomaly screens are typically undertaken. However, an increasing number of sonographers have suggested that limb observance should be performed during first trimester, when nuchal translucency is tested^15^. McEwing *et al*.^16^ reported the earliest diagnosis of osteogenesis imperfecta type II at 9 weeks and 3 days. Additionally, Blitz MJ *et al*. reported a case of ectrodactyly at 13 weeks^17^. It is clear that three-dimensional ultrasonography and transvaginal sonography can provide a more detailed evaluation of suspected abnormalities. Erashy *et al*.^18^ reported that the ability to identify limb buds was only 68.7% in cases where transabdominal sonography was used alone, whereas both limb buds were seen in 97.7% of cases when transvaginal sonography was used. As three-dimensional ultrasonography can improve the understanding of the fetal condition and provide additional data due to the simultaneous evaluation in 3 orthogonal planes, it is prevalent in first-trimester evaluation. Furthermore, most clinicians believe that it can improve the low detection rate of limb abnormalities that is associated with the use of two-dimensional ultrasonography during the first trimester.

Nonetheless, we conclude that using the metacarpal bone plane to screen for severe forearm and hand abnormalities is a quick, easy, and efficient method with high sensitivity and precision. At the same time, we note the following points with respect to the method. 1. It is important to choose an appropriate gestational age: the ossification center is initially shaped by 12 gestational weeks, and the echo of fetal limbs is visible by the second trimester. However, in the third trimester, ultrasonoscopy of fetal limbs is not satisfactory, due to the relatively larger size of the fetus, the reduction in the volume of amniotic fluid, and the visual obstruction by the fetal body or head. In our study, we chose 16–34 gestational weeks. 2. The limb should be scrutinized in a fixed order: a systematic continuous sequence approach should be used to carefully observe the limb, beginning at the proximal upper limb and continuing to the far end along the long axis, until the palm is reached. 3. Interference factors should be avoided: if the forearm and hand are blocked by the fetal head, the uterine wall, or the placenta, the pregnant woman should be asked to get up and walk around. Subsequently, the examination may be continued after the fetus has changed its position. 4. Other associated abnormalities should be sought if the fetus shows forearm and hand malformation: forearm and hand malformation are often accompanied by other deformities, such as those of the nervous system, heart, or urinary system. Similarly, when deformities in other systems are found, the presence of forearm and hand malformations should also be assessed.

In conclusion, using ultrasound to observe the metacarpal bone plane so as to detect serious forearm and hand malformation, with the exception of simple polydactyly and dactylion deformity, is effective and feasible. The metacarpal bone plane should be included in routine observance during obstetric ultrasound examinations, and this method should be employed more widely in primary hospitals.

## Additional Information

**How to cite this article:** Zhou, L. *et al*. Metacarpal Bone Plane Examination by Ultrasonography for the Diagnosis of Fetal Forearm and Hand Deformity. *Sci. Rep.* **7**, 42161; doi: 10.1038/srep42161 (2017).

**Publisher's note:** Springer Nature remains neutral with regard to jurisdictional claims in published maps and institutional affiliations.

## Figures and Tables

**Table 1:** The value of the second to fifth metacarpal plane in detecting severe forearm and hand abnormalities.

		Autopsy or newborn display		
		positive	negative	Total
Abnormalities on sonography	positive	134	1	135
	negative	4	20,000	20,004
Total		138	20,001	20,139
